# Annual costs of chronic obstructive pulmonary disease in Finland during 1996–2006 and a prediction model for 2007–2030

**DOI:** 10.1038/npjpcrm.2015.15

**Published:** 2015-03-26

**Authors:** Fredrik Herse, Toni Kiljander, Lauri Lehtimäki

**Affiliations:** 1 Nordic Healthcare Group, Helsinki, Finland; 2 Department of Respiratory Diseases, Terveystalo Hospital, Turku, Finland; 3 School of Medicine, University of Tampere, Tampere, Finland; 4 Allergy Centre, Tampere University Hospital, Tampere, Finland

## Abstract

**Background:**

Chronic obstructive pulmonary disease (COPD) is a major burden for the health care system, but the exact costs are difficult to estimate and there are insufficient data available on past and future time trends of COPD-related costs.

**Aims:**

The aim of the study was to calculate COPD-related costs in Finland during the years 1996–2006 and estimate future costs for the years 2007–2030.

**Methods:**

COPD-related direct and indirect costs in the public health care sector of the whole of Finland during the years 1996–2006 were retrieved from national registers. In addition, we made a mathematical prediction model on COPD costs for the years 2007–2030 on the basis of population projection and changes in smoking habits.

**Results:**

The total annual COPD-related costs amounted to about 100–110 million Euros in 1996–2006, with no obvious change, but there was a slight decrease in direct costs and an increase in indirect costs during these years. The estimation model predicted a 60% increase up to 166 million Euros in COPD-related annual costs by the year 2030. This is caused almost entirely by an increase in direct health care costs that reflect the predicted ageing of the Finnish population, as older age is a significant factor that increases the need for hospitalisation.

**Conclusions:**

The total annual COPD-related costs in Finland have been stable during the years 1996–2006, but if management strategies are not changed a significant increase in direct costs is expected by the year 2030 due to ageing of the population.

## Introduction

Chronic obstructive pulmonary disease (COPD) is highly prevalent worldwide, and it is estimated to become the third most common cause of death globally by the year 2020.^1^ The prevalence of COPD in the Finnish adult population is about 4–10% depending on the definition used.^2–4^ In two recent population-based studies, the prevalence of COPD, as defined by post-bronchodilator ratio of forced expiratory volume in 1 s to forced vital capacity <0.7, was 9.4% among 21–70-year-old subjects in Northern Finland^2^ and 5.9% among 26–74-year-old subjects in Helsinki.^4^ Of the latter population, 0.6% had severe, 3.0% had moderate and 2.2% had mild COPD. These figures are roughly in line with those published internationally,^5^ but even higher figures have been reported in, e.g., Northern Sweden^6^ and Denmark.^7^ Although the age-adjusted prevalence of COPD has not changed during the last few decades in Finland,^3^ the overall incidence and prevalence of COPD may increase due to ageing of the population.

COPD is a major disease in economic terms as well. The direct annual health care costs of COPD in the European Union are estimated to be about 23.3 billion Euros.^8^ A large share of this is thought to be caused by hospitalisations due to acute exacerbations of COPD. Comorbidities related to COPD further increase the total costs, but their effects are difficult to estimate. As COPD-related health care costs are associated with the severity of the disease, early diagnosis and smoking cessation is advocated to hinder the disease progression^1^ and thereby hopefully also decrease health care costs.

New drugs, such as long-acting bronchodilators, have been introduced for the treatment of COPD during the last two decades. The use of these drugs increases the direct medicinal costs of COPD. However, as the drugs improve health status in subjects with COPD,^1^ they might in turn decrease the costs caused by outpatient visits. Most importantly, the drugs are also shown to decrease the risk for acute exacerbations and hospitalisations,^1^ and this might be an important factor in decreasing the total COPD-related costs. Further, changes in treatment protocols of hospitalised patients towards shorter in-hospital periods are likely to affect the total costs. Unfortunately, it is not well known how these factors have changed the COPD-related health care costs on a nationwide basis. The Finnish health care system with its comprehensive statistics offers a good opportunity for estimating COPD-related costs and the effects of the above-mentioned parameters on the costs.

The aims of the current study were to study the change in total national COPD-related costs in Finland during the years 1996–2006 and estimate the future annual COPD-related costs in Finland for the years 2007–2030. The study was conducted by the Nordic Health Care Group and it was funded by Boehringer-Ingelheim Finland.

## Materials and Methods

### Annual costs 1996–2006

The annual costs of COPD during the years 1996–2006 were calculated using health care registers and statistics covering the whole population in Finland (5.1 million people in 1996 and 5.3 million in 2006). The direct costs of COPD were regarded as costs caused by hospitalisations due to COPD, outpatient visits due to COPD and the costs of COPD medication. The indirect costs of COPD included sickness allowances and disability pensions due to COPD. In contrast to some other studies, we did not first identify a certain population of subjects with COPD and then add up all their health care costs from registers using their personal identity codes. Instead, we utilised national registers of different health care services or social benefits and identified all such events where COPD was labelled as the cause of that service/benefit.

#### Direct costs

##### Costs of hospitalisations and outpatient visits

The core of the Finnish health care system is public health care, which consists of primary (both outpatients and inpatients) and secondary health care (both outpatients and inpatients) services. All outpatient visits, hospitalisations and medical procedures in secondary public health care and hospitalisations in primary health care are registered with the related ICD-10 diagnosis code by the National Institute for Health and Welfare.^9^ The costs for private health care or occupational health care systems were excluded, as no register data of COPD patients treated in those sectors could be obtained.

We included in the analysis all outpatient visits, days in hospital and medical procedures with ICD-10 diagnostic codes J43 (emphysema) or J44 (COPD) as the primary cause. The unit costs in 2006 for a day in hospital and for an outpatient visit were obtained from the National Institute for Health and Welfare^10^ and are given in Table 1. The pricing of medical procedures was based on average DRG prices quoted by hospitals.^10^ The unit costs for other years were derived from these by using inflation corrections with price index on public expenditure provided by Statistics Finland.^11^

##### Medication costs

In Finland, all drugs bought from pharmacies are registered with the buyers' identity to the Finnish Statistics on Medicines,^12^ but this register does not include the diagnostic codes. However, patients with severe enough asthma or COPD gain a special reimbursement for their medication. If a person entitled to such a reimbursement buys medication, data on this can be retrieved from the registers. Subjects with asthma and COPD are not separated in this register but are considered a single group of patients with obstructive pulmonary diseases. To avoid including medication used to treat asthma, we defined COPD-related drugs as drugs for the treatment of respiratory diseases bought by persons who were entitled to a special reimbursement for obstructive pulmonary diseases and who also used inhaled anticholinergics (these were only seldom used to treat asthma in Finland during 1996–2006). The costs for these COPD-related drugs were gained from the Finnish Statistics on Medicines.^12^

#### Indirect costs

Indirect costs of COPD, i.e., the loss of productivity due to illness, were calculated on the basis of sickness allowances and disability pensions due to COPD. In Finland, all sickness allowances with the ICD-10 code of the cause are registered by the Social Insurance Institution,^13^ and disability pensions with the ICD-10 code of the cause are registered by the Finnish Centre for Pensions.^14^ The data we retrieved from the registers included the number of days on sickness allowance and the number of subjects on disability pension due to COPD (J33 or J44). These were then transformed into lost man-years, and the costs for these were calculated using the unit cost, quoted by the National Institute for Health and Welfare, of 24,600€ per year in 2006.^10^

Mortality caused by COPD was not included in the calculations of loss of productivity, as most COPD-related deaths occur at older age when the subject has already retired. We excluded also the following indirect costs that are difficult to define and calculate: impaired productivity at work while still present (presenteism),^15^ work outside of paid work, care-giving by family members and immaterial costs, the loss of productivity or decrease in retirement pensions due to mortality caused by COPD.

### Estimated annual costs for 2007–2030

The estimation model was built on an assumption that smoking history, age and sex are the major factors determining (the risk of COPD and) COPD-related costs. In this model, we did not try to estimate the actual number of subjects with COPD, but our model assumes that the fractions of active smokers and recent quitters that have COPD and cause COPD-related costs remain the same in the future.

First, we assessed the total COPD-related costs in 2006, as described in the first part of the manuscript, separately in each age group (5-year intervals) in both sexes. Second, we retrieved the percentages of active smokers and recent quitters (during the previous 10 years) separately in each corresponding age group and both sexes from the Tobacco Statistics in Finland.^16^ We then calculated in each age group and sex ‘smoking standardised COPD costs’ in 2006 by dividing the actual COPD-related costs in 2006 by the number of active smokers and recent quitters with the following weighing factors: 1 for active smokers and those ex-smokers who had quit 1–3 years ago; 0.75 for ex-smokers who had quit 3–5 years ago; and 0.5 for ex-smokers who had quit 5–10 years ago.

To calculate the COPD-related costs in each age group and gender in the years 2007–2030, we needed to calculate the numbers of smoking subjects and recent quitters in each age group and gender in each of the years. We began by calculating the percentages of active smokers and recent quitters in each age group and sex for each year from 2007 to 2030 by assuming that the fractions of active smokers and recent quitters (1–3 years ago, 3–5 years ago and 5–10 years ago) continue to change at the same rate they have changed during the years 1996–2006 in Finland based on the Tobacco Statistics.^16^ We then multiplied these percentages of smokers and recent quitters by the estimated numbers of subjects in each age group and sex retrieved from the population projection in Finland^17^ to get the actual numbers of smokers and recent quitters in each group and year. Finally, these numbers of active smokers and recent quitters in each age group, sex and year were multiplied by the ‘smoking standardised COPD costs’ in 2006 using the same weighing factors for smokers and quitters as mentioned above. These total annual COPD-related costs during the years 2007–2030 were then corrected by an assumed annual inflation factor of 2%.

## Results

### Annual costs 1996–2006

The numbers of outpatient visits, days in hospital, days on sickness allowance and sickness pensions retrieved from the registers are given in Table 2, both in absolute values and also per 100,000 inhabitants. There was a 17% increase in primary care hospital days but a simultaneous 52% decrease in more expensive secondary care hospital days. Changes in outpatient visits, days on sickness allowances and persons on disability pensions were not marked.

**Figure 1:**
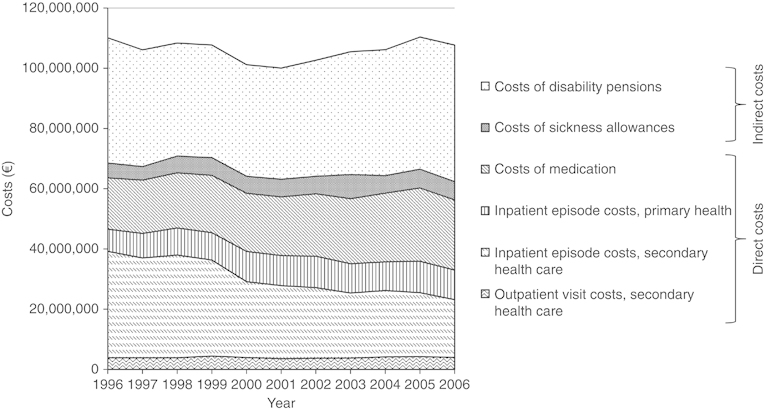
Total COPD-related costs in Finland during the years 1996–2006 retrieved from national registers and divided into major components. COPD, chronic obstructive pulmonary disease.

The time trends of key COPD-related costs in the period 1996–2006 are presented in Figure 1, and the exact numbers in total and per 100,000 inhabitants in the years 1996 and 2006 are given in Table 3. The annual total COPD-related costs in Finland varied between 101 and 110 million Euros during the period 1996–2006 and there was no obvious change (Figure 1). However, there was a slight decrease in direct costs from 63.6 to 56.3 million Euros and a similar slight increase in indirect costs from 46.5 to 51.4 million Euros during these years (Table 3). The decrease in direct costs was caused by reduced number of days in hospital in secondary care causing a marked decrease in costs of about 16 million Euros. However, this was somewhat outweighed by a simultaneous increase in medication costs of about 6 million Euros and by smaller increases in other direct costs. The rise in indirect costs was caused mainly by increase in unit costs for lost productivity, as there were no marked changes in the days on sickness leave or subjects on disability pension (Table 2).

### Estimated annual costs for 2007–2030

**Figure 2:**
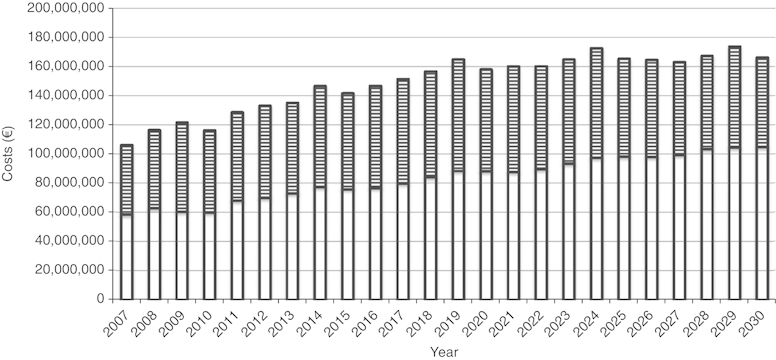
Predicted total COPD-related costs in Finland during the years 2007–2030 divided into direct (open bars) and indirect (hatched bars) costs. COPD, chronic obstructive pulmonary disease.

On the basis of our calculations, the annual COPD-related costs in Finland will increase by about 60% during the years 2007–2030 from 107 to 166 million Euros (Figure 2). The main reason for this increase is the significant ageing of the Finnish population in the years to come, as both the prevalence of COPD and especially the use of health care services increase with ageing. Even the decreasing trend in the fraction of smokers does not seem to balance out the effect of ageing. This ageing-related increase in total costs is caused almost entirely by increasing direct costs (hospitalisations, outpatient visits and medical costs). The indirect costs will not change substantially during the years 2007–2030, as increased morbidity at older age, when already retired from work, does not affect the productivity.

## Discussion

### Main findings

The main findings of the study were that the total COPD-related costs in Finland were stable at about 100 million Euros annually during the years 1996–2006, but due to the forthcoming ageing of the population the annual costs are estimated to increase by about 60% by the year 2030.

### Interpretation of findings in relation to previously published work

Cost-of-illness studies can be divided into two types: top-down studies, like this one, estimating total costs on the basis of the register data on a national level, and bottom-up studies that follow individual patients recording all their costs and then extrapolating these to national level by taking into account disease prevalence. Top-down studies generally include a limited number of different costs and they exclude underdiagnosing, whereas bottom-up studies include costs of comorbidities as well, and the prediction of total national costs is dependent on the accuracy of prevalence estimates. These issues must be taken into consideration when comparing different studies.

A Swedish top-down study^18^ estimated that in 1991 the total COPD-related cost in Sweden was about 3.2 million Euros per 100,000 inhabitants (2,784 million SEK (Swedish Krona) in total, 1 €≈10 SEK and Sweden’s population was about 8.6 million in 1991). This estimate is somewhat higher than our estimate of total COPD-related costs in Finland in the year 1996 (2.1 million Euros per 100,000 inhabitants). This difference may be, in part, explained by the fact that the Swedish study also estimated mortality-related costs and by the fact that the mean length of hospitalisation may have decreased already between 1991 and 1996 in both the countries. A more recent study from Sweden^19^ gave a much higher estimate of the total COPD-related costs, totalling 15.7 million Euros per 100,000 inhabitants (1.46 billion Euros in total; Swedish population 9.3 million in 2010), but this may be explained by the bottom-up design of the study. In Denmark in the year 2002, COPD-related costs were estimated by a top-down study^20^ to be 4.7 million Euros per 100,000 inhabitants (256 million Euros in total; Danish population 5.4 million in 2002), which is more in the same range with our study and the top-down study from Sweden.

A previous study in Finland reported that the annual costs of COPD were 194 million Euros.^21^ The difference from our result (about 100 million) may be explained by the broader spectrum of diagnostic labels included in the previous study (also J40–J42 (chronic bronchitis) and J47 (bronchiectasis)) in retrieving health care utilisation data from the registers. Furthermore, the previous study calculated hospital treatment to be caused by COPD even if COPD was not the primary diagnosis, whereas the current study included only those treatment periods in which COPD was labelled as the primary cause of hospitalisation. Including only treatment periods with COPD as the primary diagnosis may underestimate the costs caused by acute exacerbations of COPD, as some of these periods may be labelled with a diagnosis of acute bronchitis or pneumonia. On the other hand, considering that all treatment periods are caused by COPD, where COPD is included in the list of the patient’s diagnoses, surely causes overestimation of COPD-related costs, as these subjects are treated for other diseases as well. Such differences between studies make it difficult to compare the results.

A previous study^22^ has estimated that COPD-related costs in Finland have decreased by 88% from 1997 to 2007. However, the costs at different time points were retrieved from different studies with considerable differences in the calculation methods used. This severely undermines the reliability of the stated huge decrease in COPD-related costs. To our knowledge, the current study is the first using the same methods consistently in assessing the annual COPD-related costs in Finland during a wide range of years. We found that there was no obvious change in total COPD-related costs during the period 1996–2006.

Although the total COPD-related costs were stable during the years 1996–2006, there was a decrease in the direct costs and a corresponding increase in the indirect costs. The direct costs decreased mainly due to a decrease in the number of in-hospital days. This reflects changes in clinical practice regarding the treatment of acute exacerbations of COPD. The number of hospital treatment periods was about the same in 1996 and 2006, but the mean length of the treatment periods decreased, which also decreased the total number of treatment days. Our finding is in line with a recent report from Sweden^19^ in which costs of hospitalisations due to COPD decreased between the years 1999 and 2010 but there was a simultaneous increase in medication costs.

There are not many prediction studies of future COPD costs available internationally, but a recent study^23^ estimated a 53% increase in national COPD costs in the United States from 32.1 billion dollars in 2010 to 49 billion dollars by the year 2020. This is in line with our estimate of a 60% increase in COPD costs by the year 2030 in Finland.

### Strengths and limitations of this study

This study is based on national registers covering the public health care system in the whole of Finland and gives a reliable estimate on the true costs of COPD. However, our estimate of total costs is likely a conservative one because of several reasons. First, the exact reason and primary diagnosis for hospitalisation of a subject with COPD and many comorbidities is not always straightforward, and some exacerbations of COPD are certainly treated under other diagnoses, which may lead to underestimation of hospitalisation costs. Second, outpatient visits in private health care systems or in primary public health care could not be included because of lack of reliable registers. However, this is probably not a significant issue, as the costs of outpatient visits are small when compared with hospitalisations and indirect costs, and the number of visits of elderly people in private health care is very small. Third, not all patients with mild or moderate COPD have a special reimbursement for medication, and the medication costs of subjects without this reimbursement are not included in this analysis. As medication costs are higher in subjects with severe COPD, this may not be a marked flaw but leads to underestimation in any case. Furthermore, the indirect costs are always difficult to define and calculate, and we did not include, e.g., the possibility that mortality due to COPD might decrease the expenses of retirement pensions due to earlier death of subjects with COPD.

Our estimate of future costs assumes that the effectiveness of the health care system and the age-adjusted need for hospitalisation will be at the current level in the future also. However, if new treatments emerge that significantly decrease the need for hospitalisation or shorten hospitalisation periods, the increase in future costs might be smaller than estimated here. Our model on the future prevalence of COPD also assumed that the age-adjusted proportion of smokers in the population will continue to decrease at the same rate as it has in previous years. If the smoking trends deviate from the assumption either up- or downwards, this might decrease the liability of our estimate on the prevalence of COPD. However, there is a considerable latency period between changes in smoking habits and changes in COPD prevalence, and our time period for the estimate is only 24 years. Therefore, the accuracy in predicting future smoking habits is not that important for the time period of 24 years, but it would be much more pivotal for longer time periods.

A weakness of the current study, as with all prediction models, is that in predicting future COPD-related costs we needed to estimate several parameters on the basis of their time trends in the past. However, while doing this we tried to be as realistic, or even conservative, as possible.

### Implications for future research, policy and practice

In the past, the prevalence of COPD has been on the rise due to ageing of the population, and the medicine costs have also risen as new drugs have become available. However, these factors increasing the costs have been balanced out by increased efficiency of the health care system that has decreased the length of in-hospital periods. The Finnish population will be ageing much more rapidly during the next 20 years than during the years 1996–2006. The number of patients with COPD will increase especially in the older age groups, in which also the need for hospital treatment is the greatest. This is why there will be ageing-related increase in total COPD-related costs in the future, although this did not take place in the past. As the older age groups have already retired, the higher prevalence of COPD among these subjects does not cause an increase in indirect costs that are related to loss of productivity.

The ageing-related increase in COPD costs should be taken into account in Finland when planning the national health care strategy. The results can be expanded to other western countries with similar smoking habits and ageing of the population. As the streamlining of the health care system in treating exacerbations has already taken place, this is likely not a significant option in reducing the costs anymore. Instead, focus should be on smoking policy to decrease age-adjusted COPD incidence and on the management of stable COPD to decrease the risk for exacerbations.

### Conclusions

In conclusion, the annual costs of COPD in Finland have been stable during the years 1996–2006, but the costs are estimated to increase by 60% until the year 2030 mainly due to ageing of the population. A strict smoking policy is needed to decrease COPD-related morbidity and the future rise in costs, and possible new treatment options need to be assessed in terms of cost effectiveness as well.

## Figures and Tables

**Table 1:** The unit costs of health care services and cost for lost man-years due to sickness allowance or disability pension obtained from the National Institute for Health and Welfare in the year 2006

	*Unit cost, €*
Days in hospital in primary care	142
Days in hospital in secondary care	590
Secondary care outpatient visits	200
Lost man-year due to sickness allowance or disability pension	24,600

**Table 2:** Numbers of hospitalisation days, outpatient visits, days on sickness allowance and subjects on disability pension retrieved from registers in 1996 and 2006

	*1996*	*2006*
Population in Finland	5,132,320	5,276,955
Days in hospital in primary care	58,986 (1,149)	69,021 (1,308)
Days in hospital in secondary care	68,231 (1,329)	32,446 (615)
Secondary care outpatient visits	22,179 (432)	20,115 (381)
Days on sickness allowance due to COPD	59,104 (1,152)	64,354 (1,220)
Subjects on disability pension due to COPD	1,928 (38)	1,844 (35)

The numbers are in total values in Finland and per 100,000 inhabitants in parenthesis.

Abbreviation: COPD, chronic obstructive pulmonary disease.

**Table 3:** Direct and indirect costs of COPD in the whole population of Finland in the years 1996 and 2006, both in absolute values and per 100,000 inhabitants

	*1996*		*2006*	
	*Total costs (€)*	*Costs per 100*,*000 inhabitants (€)*	*Total costs (€)*	*Costs per 100*,*000 inhabitants (€)*
Outpatient visit costs, SHC	3,890,214	75,798	4,023,000	76,237
Hospitalisation costs, SHC	35,321,979	688,226	19,154,939	362,992
Hospitalisation costs, PHC	7,371,664	143,632	9,835,330	186,383
Costs of medication	17,046,441	332,139	23,268,935	440,954
Total direct costs	63,630,298	1,239,796	56,282,204	1,066,566
				
Costs of sickness allowances	4,897,021	95,415	6,074,400	115,112
Costs of disability pensions	41,592,319	810,400	45,348,966	859,378
Total indirect costs	46,489,340	905,815	51,423,366	974,489
				
Total costs	110,119,638	2,145,611	107,705,570	2,041,055

Abbreviations: COPD, chronic obstructive pulmonary disease; PHC, primary health care; SHC, secondary health care.

## References

[bib1] Global Initiative for Chronic Obstructive Lung Disease (GOLD) Global Strategy for the Diagnosis, Management and Prevention of COPD 2014 . Available at http://www.goldcopd.org/ .

[bib2] Kotaniemi J Sovijärvi A Lundbäck B Chronic obstructive pulmonary disease in Finland: prevalence and risk factors COPD 2005 2 331 339 17146998 10.1080/15412550500218122

[bib3] Vasankari TM Impivaara O Heliövaara M Heistaro S Liippo K Puukka P No increase in the prevalence of COPD in two decades Eur Respir J 2010 36 766 773 20693258 10.1183/09031936.00178109

[bib4] Kainu A Rouhos A Sovijärvi A Lindqvist A Sarna S Lundbäck B COPD in Helsinki, Finland: socioeconomic status based on occupation has an important impact on prevalence Scand J Public Health 2013 41 570 578 23599377 10.1177/1403494813484554

[bib5] Halbert RJ Natoli JL Gano A Badamgarav E Buist AS Mannino DM Global burden of COPD: systematic review and meta-analysis Eur Respir J 2006 28 523 532 16611654 10.1183/09031936.06.00124605

[bib6] Lundbäck B Lindberg A Lindström M Rönmark E Jonsson AC Jönsson E Not 15 but 50% of smokers develop COPD?--Report from the Obstructive Lung Disease in Northern Sweden Studies Respir Med 2003 97 115 122 12587960 10.1053/rmed.2003.1446

[bib7] Hansen JG Pedersen L Overvad K Omland O Jensen HK Sorensen HT The prevalence of chronic obstructive pulmonary disease among Danes aged 45-84 years: population-based study COPD 2008 5 347 352 19353348 10.1080/15412550802522635

[bib8] Gibson GJ Loddenkemper R Lundback B Sibille Y Respiratory health and disease in Europe: the new European Lung White Book Eur Respir J 2013 42 559 563 24000245 10.1183/09031936.00105513

[bib9] National Institute for Health and Welfare Care Register for Health Care 2014 . Available at http://www.thl.fi/en_US/web/en/statistics/data_collection .

[bib10] Hujanen T Kapiainen S Tuominen U Pekurinen M Terveydenhuollon Yksikkökustannukset Suomesssa Vuonna 2006 Stakes: Helsinki, Finland 2008

[bib11] Official Statistics of Finland (OSF) Price index of public expenditure [e-publication] 2014 . Available at http://www.stat.fi/til/jmhi/index_en.html .

[bib12] The Social Insurance Institution of Finland Finnish Statistics on Medicines 2014 . Available at http://www.kela.fi/tilastojulkaisut_suomen-laaketilasto .

[bib13] The Social Insurance Institution of Finland Statistics on Sickness Allowance 2014 . Available at http://www.kela.fi/web/en/statistics-by-topic_statistics-on-sickness-allowance .

[bib14] The Social Insurance Institution of Finland Statistics on pensions 2014 . Available at http://www.kela.fi/web/en/statistics-by-topic_statistics-on-pension-in-finland .

[bib15] Drummond MMethods for the economic evaluation of health care programmes. In: Sculpher M, Torrence G, O´Brian B, Stoddard G (eds). Basic Types of Economic Evaluation Oxford University Press: Oxford, UK 2005 7 25

[bib16] Official Statistics of Finland (OSF) Tobacco statistics [e-publication] 2014 . Available at http://stat.fi/til/tupk/index_en.html .

[bib17] Official Statistics of Finland (OSF) Population projection [e-publication] 2014 . Available at http://www.tilastokeskus.fi/til/vaenn/index_en.html .

[bib18] Jacobson L Hertzman P Löfdahl CG Skoogh BE Lindgren B The economic impact of asthma and chronic obstructive pulmonary disease (COPD) in Sweden in 1980 and 1991 Respir Med 2000 94 247 255 10783936 10.1053/rmed.1999.0733

[bib19] Jansson SA Backman H Stenling A Lindberg A Rönmark E Lundbäck B Health economic costs of COPD in Sweden by disease severity—has it changed during a ten years period? Respir Med 2013 107 1931 1938 23910072 10.1016/j.rmed.2013.07.012

[bib20] Bilde L Rud Svenning A Dollerup J Baekke Borgeskov H Lange P The cost of treating patients with COPD in Denmark—a population study of COPD patients compared with non-COPD controls Respir Med 2007 101 539 546 16889949 10.1016/j.rmed.2006.06.020

[bib21] Tynkkynen L Klaukka T Pietinalho A Rissanen P The costs of chronic obstructive pulmonary disease are lower than previously expected Finn Med J 2009 64 2095 2099

[bib22] Kinnula VL Vasankari T Kontula E Sovijärvi A Säynäjäkangas O Pietinalho A The 10-year COPD Programme in Finland: effects on quality of diagnosis, smoking, prevalence, hospital admissions and mortality Prim Care Respir J 2011 20 178 183 21431275 10.4104/pcrj.2011.00024 PMC6549818

[bib23] Ford ES Murphy LB Khavjou O Giles WH Holt JB Croft JB Total and state-specific medical and absenteeism costs of chronic obstructive pulmonary disease among adults aged ⩾18 years in the United States for 2010 and projections through 2020 Chest 2015 147 31 45 25058738 10.1378/chest.14-0972

